# Proposing a health-specific organizational impact framework to evaluate organizational impacts in health technology assessments

**DOI:** 10.1017/S0266462323000508

**Published:** 2023-10-11

**Authors:** Caryn Mathy, Christophe Pascal, Isabelle Bongiovanni-Delarozière, Lauriane Favez

**Affiliations:** 1 School of Engineering and Management Vaud, HES-SO University of Applied Sciences and Arts Western Switzerland, Yverdon-les-Bains, Switzerland; 2 Univ Lyon, Jean Moulin, IFROSS, CRDMS, Lyon, France; 3 Haute Autoritè de Santè, Medical and Public Health Evaluation Division, Saint-Denis la Plaine, France

**Keywords:** technology assessment biomedical, biomedical technology, organization and administration, decision making, health impact assessment, equipment and supplies

## Abstract

**Objectives:**

Health technology assessments (HTAs) have traditionally included clinical and cost-effectiveness evaluation of new health technologies (HTs). However, new HTs can generate important organizational impacts (OIs) that influence their overall value. OIs are currently not clearly identified and evaluated in HTA procedures and tools are limited. To address this issue, a comprehensive framework that allows to assess OIs of new HTs in HTAs is proposed.

**Methods:**

A working and methodological group identified the Oslo Manual 2018, 4th edition, OECD/Eurostat, on the objectives and outcomes of commercial innovations as the basis for the OIs framework for HTAs. The Oslo Manual was translated to the healthcare sector and adapted to HTA procedures through a three-step process.

**Results:**

The framework is composed of three main parts. Part I tackles the context of the evaluation, Part II the categories of impacts and the specific impacts – in total, 16 OIs were identified – and Part III the stakeholders involved. The central part of the framework is Part II, and consists of three categories of impacts: (i) on the care process, (ii) on the stakeholders’ capabilities and skills, and (iii) on society or the community.

**Conclusions:**

This framework provides a comprehensive and structured basis to document OIs of new HTs. It thus contributes to the extension of HTA evaluation criteria to other dimensions than clinical and economic aspects, that is, organizational aspects. Some of its intrinsic limitations and the questions they raise in the field for policy-makers, practitioners, and researchers are discussed.

## Background

Health technology assessments (HTAs) have the purpose to examine different dimensions of values of new health technologies (HTs). These dimensions can include “clinical effectiveness, safety, costs and economic implications, ethical, social, cultural and legal issues, organizational and environmental aspects” and sometimes other, wider implications (1). However, in practice, HTAs have predominately focused on clinical and cost-effectiveness dimensions of new HTs, often neglecting other aspects. Clinical and cost-effectiveness aspects have been evaluated through various indicators by HTA organizations, with the goal to determine the value of each new HT evaluated and make recommendations (e.g., reimbursement decisions). Considering the need to ensure high quality of care and the sustainability of healthcare systems and to control patient costs, these elements are undisputable. However, they do not allow all of the impacts of innovations to be evaluated (2–7). Indeed, HTs such as drugs, medical devices (MDs) or professional practices, can have impacts beyond strict diagnostic, therapeutic, disability, or economic gains. New HTs can deeply affect the global organization of care and its stakeholders at all levels (i.e., individuals, health organizations, health system, and society). For instance, the use of rapid antigen COVID-19 tests during the COVID-19 pandemic shows that the impacts of new HTs can be major, sometimes structural, and even systemic (8). These tests impacted, for instance, individuals (i.e., allowing people to test at home and to self-isolate when appropriate) but also nursing homes residents and staff (e.g., allowing quick isolation of residents), relieving the pressure on overworked labs and hospitals, and on the overall health system, at a critical point of the COVID-19 pandemic.

There have been several publications showing the relevance of evaluating organizational impacts (OIs) in HTA procedures – which can range from increased need for staff supervision or training after the introduction of a new HT, to changes in the roles of a multidisciplinary team, to modifications of care pathways or specific organizational investments (2;3;6;7;9–14). Yet, OIs are rarely evaluated by HTA organizations, partly because there are no dedicated guidelines that provide framework, methods, or tools to guide their assessment (15). Our previous research showed that some HTA organizations were indirectly evaluating OIs in their clinical or economic assessments (15). Yet, a review of the literature in the healthcare sector as well as a review of the existing work carried out by HTA organizations (i.e., agencies, bodies, institutions, or networks) found no conceptual definition of OIs and no comprehensive evaluation framework (15). The review also identified the European Network for Health Technology Assessment (EuNetHTA) Core Model as the most advanced OI evaluation model (16). The organizational domains of this model include five topics, each containing two to six questions to be answered, resulting in a total of 15 issues to be addressed (16). These arguably represent the most important organizational issues to be evaluated. However, this model does not provide a conceptual definition of OIs and is not exhaustive, and therefore needs to be adapted to the diversity of HTs (16). Indeed, further OIs not included in the EuNetHTA model can be identified when evaluating various HTs. It is problematic because using this model could mean missing out on some important OIs – positive or negative – and hence potentially misunderstanding the real value of a new HT. As such, it was concluded that the tools for analyzing and evaluating OIs were incomplete (15). Faced with these limitations, there is a need to investigate if a more comprehensive model outside of the health field could be used to facilitate the evaluation of OIs in the context of HTAs.

For the purpose of this work, and given the lack of agreed-upon conceptual definition that can be used, an OI in the context of an HTA is defined as “an effect, consequence, result, or repercussion, created by HTs on the characteristics and functioning of an organization or a set of organizations (understood as individual or collective stakeholders) involved in the care or life course of users” (17). Furthermore, the following three points should be noted: (i) OIs (and their indicators) can be understood through the resources needed to implement HTs (e.g., requires training or clinical education of the patient) or the changes involved in its deployment (e.g., leads to a change in the skillset of a health professional), (ii) OIs may be immediate or delayed, depending on whether the HT is in the learning phase or in the “routine” phase; it is therefore necessary to specify the position considered in the deployment cycle of the HT evaluated, and (iii) it should be specified whether the impact is temporary or permanent, and positive or negative; these should be reflected in the evaluation of the value associated with the implementation or deployment of an HT (i.e., added or reduced value). Finally, stakeholders affected by OIs are defined as follows: “A stakeholder is deemed to be any individual or legal entity having an interest in the care or life pathway (of the patient). This may be a healthcare professional, the patient, a carer or accompanying person, a healthcare institution, a healthcare manufacturer or any other stakeholder involved in the delivery of care or services (transport, services and distributors of equipment in particular)” (17).

Given the relevance of assessing OIs of new HTs, the French HTA agency, that is, the “Haute Autorité de Santé” (HAS), decided to include them as part of their strategic project of 2018–2024 with the aim to ultimately support the evaluation of OIs as part of their HTA procedures (18). Thus, the HAS supported this research to develop a framework of OIs that could be used to guide their comprehensive assessment of HTA procedures. As a result, the objective of this study was to propose a comprehensive framework that allows to assess OIs of new HTs in the context of HTAs.

## Methods

Based on a previously conducted literature review (15), we first searched for a pragmatic, relevant, and methodologically strong framework from other fields of economics outside of the health-specific literature, where OIs of innovations can be relevant to the health sector. Second, we adapted the identified framework to use as a comprehensive guide in the assessment of OIs as part of an HTA.

This research was led by a working group that consisted of two academics: one HAS project manager specialized in health economics and health services management, and four HAS experts specialized in evaluation of new HTs. A methodological guidance group supported the working group by providing methodological guidance and scientific support, and validated the findings. The methodological guidance group consisted of 10 experts (external to the HAS) with research backgrounds in health engineering, public health, health services management, health economics, and patients’ rights. The working group and the methodological guidance group exchanged (in written as well as in meetings) at each stage of the research. Furthermore, the methodological guidance group was officially consulted four times during 4-day meetings throughout the whole period of the research, allowing the methodological guidance group to counsel, react, and suggest amendments. In addition, a report of each meeting was systematically distributed, and the members had the possibility to make remarks or additions at any time. The work was then submitted to and validated by the three specialist committees of the HAS and its Board.

The working group identified that the Oslo Manual (4th edition), developed jointly by the Organization for Economic Co-operation and Development (OECD) (a reference organization in the field of innovation assessment) and Eurostat (19), was the most robust and comprehensive manual available to identify and assess OIs in all domains (e.g., health, economics, environmental). The Oslo Manual is a key element of a series of measurement manuals produced by the OECD under the title “Measuring Science, Technology and Innovation Activities” (19). This work, produced by the OECD and Eurostats, is a synthesis of several decades of research and analysis by international experts. The Oslo Manual addresses the need to reflect on the functioning of innovation systems beyond a description of the efforts made to invest in new knowledge, or the number and characteristics of patented inventions (19). The Oslo Manual deals with innovations in a broad sense: the considerations are equally applicable to product, process, organizational or, for example, marketing innovations. Innovations are considered to be changes that involve a significant degree of novelty for the organization, for example, they lead to an improvement in processes and as such, innovations include new HTs (e.g., medications, MDs) (19). Chapter 8, Table 8.1 of the Oslo Manual entitled “Innovation objectives and outcomes for measurement, by area of influence” specifies four areas of influence of impacts of innovations in companies: (1) market for the firm’s products, (2) production and delivery, (3) business organization, and (4) economy, society or environment (19). This table is reproduced in detail in Table 1. The Oslo Manual defines several important concepts regarding impacts. In particular, it mentions that the “objectives” and “outcomes” of new technologies can be interpreted as “expected impacts” and “achieved impacts” respectively. Although the Oslo Manual’s terminology is focused on standard economics markets (i.e., profit organisations) and buisness strategy, its authors explicitly argue that “general definitions and concepts of innovation [are] applicable to all four economic sectors (i.e., Business, Government, Non-profits serving households, and Households)” (19), which includes the health domain and healthcare organizations (e.g., public or private hospitals, physicians and nurses regardless of their place of work, patients and caregivers), thus making the Oslo Manual an appropriate basis to identify OIs of HTs.

**Table 1.** Reproduction of Table 8.1., Chapter 8 of the Oslo Manual 2018 entitled « Innovation objectives and outcomes for measurement, by area of influence »

Markets for the firm’s products
Upgrade goods or services
Expand the range of goods or services
Create new markets
Enter new markets or adapt existing products to new markets
Increase or maintain market share
Increase the reputation, brand awareness, or visibility of goods or services
Comply with market regulations
Adopt standards and accreditation
Production and delivery
Upgrade outdated process technology or methods
Improve quality of goods or services
Improve flexibility for producing goods or services
Increase the speed of producing goods or delivering services
Reduce labor costs per unit of output
Reduce material, energy costs, or operating costs per unit of output
Reduce time to market
Business organization
Improve capabilities for absorbing, processing, and analyzing knowledge
Improve sharing or transfer of knowledge with other organizations
Improve the efficiency or function of the firm’s value chain
Improve communication within the firm
Improve or develop new relationships with external entities (other firms, universities, etc.)
Increase business resilience and adaptability to change
Improve working conditions, health or safety of the firm’s personnel
Implement a new business model
Contribute to the development of standards
Economy, society, or environment
Reduce negative environmental impacts /deliver environmental benefits
Improve public health, safety, or security
Improve social inclusion
Improve gender equality
Improve quality of life or well-being
Comply with mandatory regulations
Comply with voluntary standards

*Note*: Table 8.1 from the Oslo Manual, 4th edition, reproduced with the permission of the publisher (19). In Step 1, it was assessed that in the context of HTAs, certain OIs did not make sense, such as “Increase the reputation, brand awareness, or visibility of goods or services”. Such impacts were removed from the final framework. In addition, some impacts were combined into a single impact in the final framework for the sake of simplification, for example, “Improve social inclusion” and “Improve gender equality” became “Impact on social inequalities or accessibility to care”.

Therefore, after an assessment from the working and methodological guidance groups, it was concluded that the elements from the Oslo Manual were transposable to an assessment of OIs of innovative technologies in the field of healthcare, although it required some adaptations for the context of HTAs. The list of impacts of innovations by area of influence was the starting point for a 3-step process executed by the working group to create an OI framework for HTAs. All the work took place in French. Step 1 aimed to check and make amendments to the formulation of the elements (e.g., terms, concepts) of Table 8.1. of Chapter 8 of the Oslo Manual so it could be transposed to the health sector, specifically related to HTAs (Table 1). Step 2 aimed to verify that the impacts were pertinent once transposed to the context of HTAs and irrelevant impacts were discarded. Step 3 aimed to verify the completeness of the various OIs retained. To achieve that, we used a variety of examples of innovative HTs (i.e., drugs, MDs, diagnostic and therapeutic procedures) that had been clinically and economically evaluated in HTAs by the HAS in the previous 3 years (e.g., introduction of a chirurgical robot, oral at-home chemotherapy instead of injectable hospital-based chemotherapy, active implantable medical devices, telemonitoring, CART-T cells therapy). In these cases, OIs had either been claimed by the manufacturer, or the HTA agency had suspected that the HT evaluated would have OIs, but these were not taken into account at the time of the assessment because there was no tool to do it. We checked that all the examples fitted in the OIs retained and that no OI was missing. Formulations were improved when needed. These steps led to the creation of an OI framework for new and innovative HTs, covering all types of OIs, specifically targeted to the context of HTAs. The framework was first created in French and then translated into English.

## Results

The work took place between March 2019 and July 2020, that is, over a period of 16 months. Following the steps described in the methods, the categories of impacts and the impacts are summarized in a framework, see Table 2. It is composed of three main parts (17):

- Part I is about the *context of the evaluation*. The objective of Part I is to specify the context of the new HT evaluated in relation to the existence of a conventional care solution (i.e., existence or lack of clinically relevant alternative).
- Part II concerns the *categories of impact*s *and the impacts*. The purpose of Part II is to specify the categories of impacts and impacts used to document OIs. It is therefore the central part of the overall framework design. It is structured into three categories of impacts in which a total of 16 impacts have been defined. The impacts aim to be exhaustive and may be cumulative for a given HT.
  - *Category of impacts 1* addresses the OIs of HTs on the care process. It examines the impacts that have a direct effect on the components of the care process. It includes the sequence of activities performed as part of the patient’s care pathway to prevent, maintain, or improve health. Examples of OIs in this category are, for example, whether and how the new HT modifies process timing or content (e.g., a treatment every month vs. every 6 months) or whether and how it modifies the type of staff involved in the process (e.g., a healthcare assistant or a registered nurse needed to supervise a treatment).
  - *Category of impacts 2* is about the OIs of HTs on the competencies, capacities, and skills required of the stakeholder to implement the care process (organizational capacities, sharing of competencies and skills, working conditions, financing, etc.) and to perform their tasks towards an effective and efficient implementation and deployment of the new HT. This category also tackles the efficient and effective combination and coordination of stakeholders’ resources and capabilities at the different stages of the life pathway or care process of the patient. It reflects the complex interactions between the resources and skills of the stakeholders as they are used in the care process. Examples of OIs in this category of impacts are, for example, whether and how the HT modifies the ability to share and transfer skills (e.g., between physicians and nurses, therapeutic education and self-management skills of chronic patients in telemonitoring settings), or whether and how it modifies the scheduling and planning capacities for healthcare services or the patient (e.g., intravenous therapy in hospital versus subcutaneous therapy at home and its impacts on the training of healthcare staff on telemonitoring).
  - *Category of impacts 3* is about the OIs of HTs on society or the community. It refers to a more general level of analysis than the previous two categories and focuses on the impacts of HTs on the general population. It includes impacts that do not directly concern the process of care, the care and care pathway of the patient, and/or the stakeholders involved. They can be considered as indirect effects at the macro level. Examples of impacts are, for example, whether and how the HT impacts the community in terms of health and safety (e.g., Covid-19 antigen self-tests allowing to limit infection risks to society) or whether and how it impacts social inequalities or accessibility to care (e.g., at-home treatments allowing to treat patients in areas with limited access to healthcare professionals or facilities).
- Part III is about the stakeholders involved. The objective of Part III is to specify the stakeholders concerned by the OIs. For each impact, it is necessary to stipulate the stakeholder(s) affected by each OI. Multiple and varied stakeholders may be affected by a single OI. Whenever necessary, the stakeholder(s) should be specified, including within an organization: for example, in a hospital, it might be the surgical or medical pathology department, or the day hospital. Impacts can be found within the same organization, but also between organizations, or between healthcare professionals, patients, or caregivers.

**Table 2.** Organizational impact framework for health technology assessment (HAS (8))

Part I	Part II		Part III
***Context assessment***	***Category of impact 1***	***Impacts***	***List of stakeholders involved/concerned by impact***
Is a conventional care solution available?	Impacts of the HT on the care process	1.1 Modifies times before initiation of the process	…
	*This category of impacts accounts for the sequence of activities carried out in the patient’s life and care pathway*	1.2 Modifies process pace or duration	…
Yes: The HT changes the existing conventional care (i.e., need met, existence of clinically relevant alternative)		1.3 Modifies process timing or content	…
		1.4 Modifies number or type of staff involved in the process (quantitative view of human resources)	…
No: The HT creates conventional care (i.e., unmet need, lack of clinically relevant alternative)		1.5 Modifies the type or frequency of use of products, devices, materials, equipment, infrastructures, and information systems used in the process (in terms of material or digital resources)	…
		1.6 Modifies the quality and safety of the environment or context in which the process takes place	…
	***Category of impact 2***	…
	Impacts of the HT on the capabilities and skills required of stakeholders to implement the care process	2.1 Modifies the stakeholder’s required skills (knowledge, know-how, and social skills) and expertise associated with the delivery or provision of care	…
	*This category of impacts includes organizational capabilities, skills and sharing of skills, working conditions, funding, etc.*	2.2 Modifies the ability to share and transfer skills, knowledge, and know-how with other stakeholders	…
		2.3 Modifies scheduling and planning capacities for healthcare services or the patient or care giver	…
		2.4 Modifies scheduling and planning capabilities between care structures or combinations of stakeholders	…
		2.5 Modifies stakeholders’ working or living conditions	…
		2.6 Modifies the terms, nature, or source of stakeholders’ funding	…
	***Category of impact 3***		…
	Impacts of the HT on society or the community	3.1 Impact on community in terms of health and safety	…
	*This category of impacts is a more general level of analysis and focuses on the impacts of the HT on the general population*	3.2 Impact on social inequalities or accessibility to care	…
		3.3 Impact on social or work relationships or in terms of society as a whole	…
		3.4 Impact on environmental footprint	…

*Note*: This is the proposed organizational impact framework for health technology assessments (HTAs) (17).

HT, health technology; HTA, health technology assessment.

Table 3 provides two examples of new HTs (i.e., transcatheter aortic valve implantation (TAVI) and direct antiviral agents (DAAs)) and their assessment through the OI framework. These two illustrative examples were part of the several examples that were used to test the framework. The framework was not adapted upon testing these examples, because all relevant impacts were deemed to have been identified through the framework by the working group and the methodological guidance group.

**Table 3.** Two examples of new HTs and how the OIs framework is used: TAVI and DAAs

New HT (MD): TAVI to replace aortic valve			
**Part I: context assessment**	**Is a conventional care solution available?**	**Part III: Stakeholders**	
Yes or No?	Yes – for the group of patients previously eligible for surgery: the HT changes the existing conventional care for patients eligible for surgery No – for the group of patients not previously eligible for surgery: the HT creates a conventional care solution for patients ineligible for surgery		
**Part II: Impacts**			
*Category 1*			
1.1.	Increase in the number of patients eligible for this less risky technique	Patients	Health system
1.2.	Less invasive surgical technique: reduced surgery time, reduced length of hospital stay post-surgery, reduced rehabilitation care	Patients	Hospital staff
1.3.	No need for convalescence and rehabilitation	Patients	Insurance companies
1.4	Requires the involvement of an interventional cardiologist	Hospital	Hospital staff
1.5	A TAVI is required	Industrials	
1.6	Can be implemented in a hybrid room allowing minimally invasive surgical procedures of interventional cardiology under medical imaging	Hospital	Hospital staff
*Category 2*			
2.1.	Specific training of health professionals in this specific surgical technique	Surgeons	Cardiologists
2.2	Need for close collaboration between interventional cardiologists and cardiac surgeons. Multidisciplinary consultation meetings to define eligible patients	Cardiologists	Hospital staff
2.3	Significant reduction in the duration of the surgery Reduction in post-intervention length of stay Reduction in length of rehabilitation care Increase in the number of patients treated	Hospital	Insurance companies
2.4	N/A		
2.5	Earlier return to home	Patients	Informal care givers
2.6	N/A		
*Category 3*			
3.1.	N/A		
3.2	Limited to a small number of specialized centers where a large volume of intervention is performed	Cardiovascular surgeons	Specialized centers
3.3	N/A		
3.4	N/A		

New HT (drug): DAAs for treatment of HCV			
**Part I: context assessment**	**Is a conventional care solution available?**	**Part III: Stakeholders**	
Yes or No?	Yes: the HT changes the existing conventional care and increase the number of eligible patients		
**Part II: Impacts**			
*Category 1*			
1.1.	Targeted population screening: early treatment of HCV-positive patients	Target population	Insurance companies
1.2.	Decrease of the total treatment duration Decrease in care due to the significant reduction in side effects Decrease in care needs for patients in advanced stages of the disease (fibrosis, cirrhosis, hepatocarcinoma)	Patients	Insurance companies
1.3.	Total reorganization of the care pathway: no longer hospital-centered (hospital-based hepatologists), but focus on outpatient care provided by general practitioners	Patients	Hospitals
1.4	Involvement of general practitioners in prescribing and monitoring eligible patients	General practitioners	Specialists
1.5	Substitute of an injectable treatment by an oral treatment	Patients	Nurses
1.6	Availability of treatment in pharmacies	Pharmacies	
*Category 2*			
2.1.	Training of general practitioners in the use of DAAs and patient follow-up	General practitioners	
2.2	Simplified care pathway and modification of the ability to share and transfer skills: prescription by a general practitioner instead of a specialist	Specialists	General practitioners
2.3	Impact the planning capabilities: - for hospitals: decrease the size of the patient list, occupancy rate and unscheduled requests - for general practitioners: increase the flow of patients Increase in the yearly total number of patients of general practitioners and decrease for specialists	Hospitals	Ambulatory healthcare staff
2.4	Better regulation of patient flows from the hospital (patients at the most advanced stage of the disease) to primary care settings in the city Improved coordination between healthcare professionals	Hospitals	Ambulatory healthcare staff
2.5	Decrease of sick leave during the treatment due to the absence of side effects Access to treatment for HIV co-infected patients awaiting transplantation	Insurance companies	Patients
2.6	Evolution of care expenditure from the hospital to the city	Insurance companies	Patients
*Category 3*			
3.1.	HCV eradication policy	Health system	
3.2	N/A		
3.3	Positive societal externalities	Society	
3.4	N/A		

*Note*: We have limited the number of stakeholders per impact to two. TAVI: implantation of a biological aortic valve percutaneously through the femoral artery, which is an alternative to conventional surgery under general anesthesia, with opening of the chest and placement of extracorporeal circulation. DAAs: used in the treatment of chronic HCV, achieve cure rates of more than 95 percent combined with very satisfactory safety profiles compared to previous treatments, and make it possible to eliminate HCV.

DAAs, direct antiviral agents; HCV, hepatitis C virus; HIV, human immunodeficiency virus; HT, health technology; MD, medical device; N/A, not applicable; TAVI, transcatheter aortic valve implantation.

## Discussion

This article aimed to present a comprehensive framework to assess OIs of HT innovations in the context of HTAs. This clear and dedicate tool could be used to facilitate the consideration of OIs in common practice by allowing HTA agencies to identify and describe them. The framework consists of three parts covering the context of the evaluation of the new HT, categories of impacts, and the potential impacts of each HT (i.e., impacts on the care process, on the capabilities and skills required to implement the care process, and on society and the community) as well as the stakeholders involved. This framework is a practical tool that can be used by manufacturers and large buyers (e.g., HTA organizations, hospitals, and insurance companies) to guide the evaluation of OIs within HTA procedures. It is a first step towards the documentation of OIs of innovations in the health domain, and as such contributes to the consideration of an important aspect of HTAs (1). This OIs framework needs further development (e.g., in terms of quantifying identified OIs and attributing them an economic value, when possible) and it will require a learning process in order for the different stakeholders to make it their own. Its use will lead to the identification of certain limits and will probably induce an evolution. In the discussion hereafter, we discuss how the framework might be used by HTA agencies, as well as some of the intrinsic limitations of the framework and the questions they raise in the field for policy-makers, practitioners, and researchers.

The proposed framework can be used by HTA agencies in different practical ways. For instance, an HTA agency could require manufacturers that submit a reimbursement claim for a new HT (such as one of those presented in Table 3) and state that the HT has several OIs, to clearly identify and describe them using the framework. Manufacturers claiming OIs could be asked to list the OIs, or the most important OIs, using the framework, and even to quantify them when indicators exist (or possibly suggest measurement tools). This might contribute to a better and more holistic understanding of the consequences of a new HT (1) and might reduce blanket statements and increase the industry’s accountability. HTA agencies could also specify that any negative OI must be disclosed, which could impact negotiations and alter reimbursement decisions. For instance, Table 3 discloses positive and negative OIs for TAVI and DAAs and stakeholders concerned with these impacts.

Regarding limitations, first, this framework allows to identify OIs and to document them, but it does not allow to quantify the OIs (e.g., through indicators) or to value them. It is an informational tool and should not be considered a scoring tool. Consequently, it is not expected that all the types of impacts proposed in the framework will be addressed by the industry when requesting an assessment. However, the question regarding scoring OIs remains open: could or should OIs scoring be done? If so, would each OI have the same weight as another? If not, what weight should be given to each OI? By extension, this raises the question of the place given to OIs in relation to clinical and economic impacts by HTA agencies in the decisions to reimburse. Despite these questions, the French HTA agency has already included this OIs framework in its HTA procedures for new MDs: companies must use it to describe and document their claims of OIs in HTA procedures (20). This should increase the consideration of OIs in the assessment of the value of new HTs entering the market, not only in France but in other countries with HTA agencies. Indeed, it should be possible to use the framework developed across settings without modifications to document OIs, but its future use across different health systems will inform its generalizability.

Beyond the question of whether OIs should be scored, quantifying OIs, particularly those relating, for example, to the development of skills, the ability to share information or to coordinate patient care pathways, is difficult because such impacts are often intangible; we do not know how to quantify them. Indeed, these impacts refer to the intellectual capital mobilized in healthcare organizations, sometimes also identified under the term intangible assets in the disciplines of accounting or financial evaluation (21). Some of the impacts relating to intellectual capital are now more easily quantifiable, because of, for example, information systems that allow to measure the number of connections to a patient record, thus providing one way to quantify the improvement in information sharing. However, even when there are relevant indicators to measure these impacts, the most accurate ones might not be routinely collected in healthcare settings (i.e., the data is not available to assess the impacts). As such, researchers should tackle methods to quantify and value OIs. This is especially challenging regarding category of impacts 2 which concerns many intangible impacts. Research on how to quantify such impacts, for example using tools of reported outcome measures from healthcare professionals or other stakeholders, building on what is done for patient-reported outcome or experience measures, should be further investigated.

Moreover, many other impacts, such as changes in skills or knowledge, are difficult to objectify directly. They cannot be observed and understood in the same way as financial and material capital (21). The alternative is to identify them indirectly, through the financial valuation of companies. Traditionally, experts in financial valuation consider that the accumulation of these assets is reflected in firms’ market value, as revealed by voluntary transactions among buyers and sellers of the firms’ financial securities (22). However, this method poses two other problems. First, this solution encompasses all intangible assets and not only those related to a new HT. Second, many healthcare organizations are not valued in the financial market and it is therefore not possible to estimate their intangible assets. Alternatively, one can consider that these assets should provide real returns in the form of higher output. Thus, a production function framework should reveal that firms that have put in place more of these intangibles saw greater output in subsequent years, after accounting for standard inputs (such as capital, labor, and materials) (19). This solution raises an additional question concerning the valuation of OIs, which is the third limitation of the framework. Indeed, this research did not aim to provide or inform on the methodology that should be used for the economic valuation of OIs. Yet, this is a major question and there is a need to be extremely careful, especially regarding the management of double counting: if these impacts are, on the one hand, valued directly (considered as OIs) and, on the other hand, also valued indirectly via their results (considered as economic impacts), then this solution generates double counting (23;24). In this respect, we note that the CoreModel developed by EunetHTA does not specify the methodology to be used for the valuation of OIs (16). Research is also needed on whether and which OIs should be valued, and the appropriate methodology to do that, taking into account the issue of double-counting.

Finally, describing, quantifying, and valuating OIs requires many more points of attention than those briefly mentioned above, such as whether the impact is positive or negative, whether it is individual or collective, and the timing of the assessment (i.e., at what point in the technology’s deployment the impacts should be measured). These impacts can be mixed, and there can be positive and negative OIs for a single new HT. Furthermore, other aspects are being discussed as potentially important HTA elements, beyond organizational aspects, such as ethical impacts of new HTs or the use of Real-World Data to assess new HTs (25;26). The evaluation of such considerations should not overlap with the OIs identified in this framework. All these need to be considered for the evaluation of the real value of a new HT and such points of attention must be tackled and solutions must be offered.

## Conclusion

The framework presented in this article provides a basis for the scientific community and HTA organizations to supplement the clinical and economic aspects of HTA evaluation criteria with OI dimensions. This framework allows to circumscribe the perimeter of OIs while offering a synthetic but comprehensive list of the elements that are considered OIs. This framework should be applicable to whatever the HT evaluated is and thus make it possible to rely on a unique and comprehensive tool.

## Data Availability

Data sharing not applicable to this article as no datasets were generated or analyzed during the current study.
